# Anisotropic and age-dependent elastic material behavior of the human costal cartilage

**DOI:** 10.1038/s41598-021-93176-x

**Published:** 2021-06-30

**Authors:** Matthias Weber, Markus Alexander Rothschild, Anja Niehoff

**Affiliations:** 1 Institute of Legal Medicine, Faculty of Medicine, University of Cologne, 50823 Cologne, Germany; 2 Institute for Forensic Sciences, Landeskriminalamt Nordrhein-Westfalen, 40219 Düsseldorf, Germany; 3 Institute of Biomechanics and Orthopaedics, German Sport University Cologne, 50933 Cologne, Germany; 4 Medical Faculty, Cologne Center for Musculoskeletal Biomechanics (CCMB), University of Cologne, 50931 Cologne, Germany

**Keywords:** Soft materials, Materials science, Tissues

## Abstract

Compared to articular cartilage, the biomechanical properties of costal cartilage have not yet been extensively explored. The research presented addresses this problem by studying for the first time the anisotropic elastic behavior of human costal cartilage. Samples were taken from 12 male and female cadavers and unconfined compression and indentation tests were performed in mediolateral and dorsoventral direction to determine Young’s Moduli E_C_ for compression and E_i5%_, E_i10%_ and E_imax_ at 5%, 10% and maximum strain for indentation. Furthermore, the crack direction of the unconfined compression samples was determined and histological samples of the cartilage tissue were examined with the picrosirius-polarization staining method. The tests revealed mean Young’s Moduli of E_C_ = 32.9 ± 17.9 MPa (N = 10), E_i5%_ = 11.1 ± 5.6 MPa (N = 12), E_i10%_ = 13.3 ± 6.3 MPa (N = 12) and E_imax_ = 14.6 ± 6.6 MPa (N = 12). We found that the Young’s Moduli in the indentation test are clearly anisotropic with significant higher results in the mediolateral direction (all *P* = 0.002). In addition, a dependence of the crack direction of the compressed specimens on the load orientation was observed. Those findings were supported by the orientation of the structure of the collagen fibers determined in the histological examination. Also, a significant age-related elastic behavior of human costal cartilage could be shown with the unconfined compression test (*P* = 0.009) and the indentation test (*P* = 0.004), but no sex effect could be detected. Those results are helpful in the field of autologous grafts for rhinoplastic surgery and for the refinement of material parameters in Finite Element models e.g., for accident analyses with traumatic impact on the thorax.

## Introduction

Human rib cartilage or costal cartilage connects the ribs to the sternum. It is a fibrous tissue that belongs to the hyaline cartilage and consists of chondrocytes and the extracellular matrix, which is mainly composed of water, collagen fibrils and proteoglycans. Hyaline cartilage is often described as a multiphasic material with a porous elastic solid phase which is predominantly formed by strong type II collagen fibrils enmeshed with proteoglycans, a fluid phase mainly consisting of water and a third phase of dissolved ions^1–3^. When pressurized, the interstitial fluid flows out of the solid phase and gets drawn back into it, when the pressure is removed. This material composition provides compressive resilience and a viscoelastic material behavior.

Very little is known about the elastic material properties and the structure of the collagen network of costal cartilage. The ultimate strength of costal cartilage has been determined in tensile tests from 2.0 to 5.6 MPa^4–6^, in compression tests to 8.3 ± 1.0 MPa^7^ and for bending from 5.7 to 24.7 MPa^4,6^. The Young’s Modulus, as a measure of material stiffness, has been measured in tension tests from 1.3 to 23.5 MPa^5,8^, in bending tests from 0.3 to 11.7 MPa^6,9^ and in unconfined compression tests from 7.7 to 133.5 MPa^9–11^. Indentation tests yielded an average instantaneous modulus from 8.7 to 12.6 MPa^12^ and a Young’s Modulus of 5.3 MPa^13^ for adult human costal cartilage. Using atomic force microscopy, the Young’s Modulus of costal cartilage was measured between 0.85 and 7.9 MPa^14^.

Studies on the influence of sex and age on the elastic properties of human costal cartilage are very limited. Guo et al.^4^ studied the influence on the tensile strength of costal cartilage of 25 female and 45 male donors of 5–25 years of age. They found the tensile strength of children (5–10 years) to be significantly higher than that of adolescents (11–17 years) and adults (18–25 years), but found no significant difference between adolescent and adults and no influence of the sex in all three age groups. Lau et al.^13^ studied the stiffness of the midsubstance of costal cartilage and found no influence based on subject age. Nevertheless, the aging process fundamentally changes the structure of costal cartilage. In the so-called ''amianthoid transformation”^15,16^, the collagens in the cartilage fuse to form fibrils with an average larger diameter up to 0.5 µm^17^ which is presumed to affect the mechanical properties. Calcification, the accumulation of calcium salts in cartilage tissue, which spreads with age, is presumably having a substantial effect on the elastic properties^18,19^.

Previous studies have addressed the arrangement of the collagen fibrils of cartilage^20–22^. The ultrastructure of adult human articular cartilage was found to be a network of fine cross-banded filaments with diameters of 10 to 15 nm. Stacey et al.^23,24^ studied the collagen fibers in human costal cartilage and describe a straw like structure, running the length of the tissue. However, although Lau et al.^13^ suggested further studies of anisotropy and changes in anisotropy with age, no work has been published yet that explores the possibility of anisotropic elastic material properties of the human costal cartilage. Furthermore, no study has yet been published comparing the material properties of the individual rib cartilages. The goal of this study is to investigate the directional material behavior of the rib cartilage, the material properties of the individual rib cartilages and also the influence of age and sex. The direction-dependent material parameters play an important role in the computer-aided simulation of accident events and the resulting injuries^25–27^, for example in traffic accidents. In addition, these parameters are required in the forensic examination of tool marks on tissue.

## Materials and methods

### Samples

**Figure 1:**
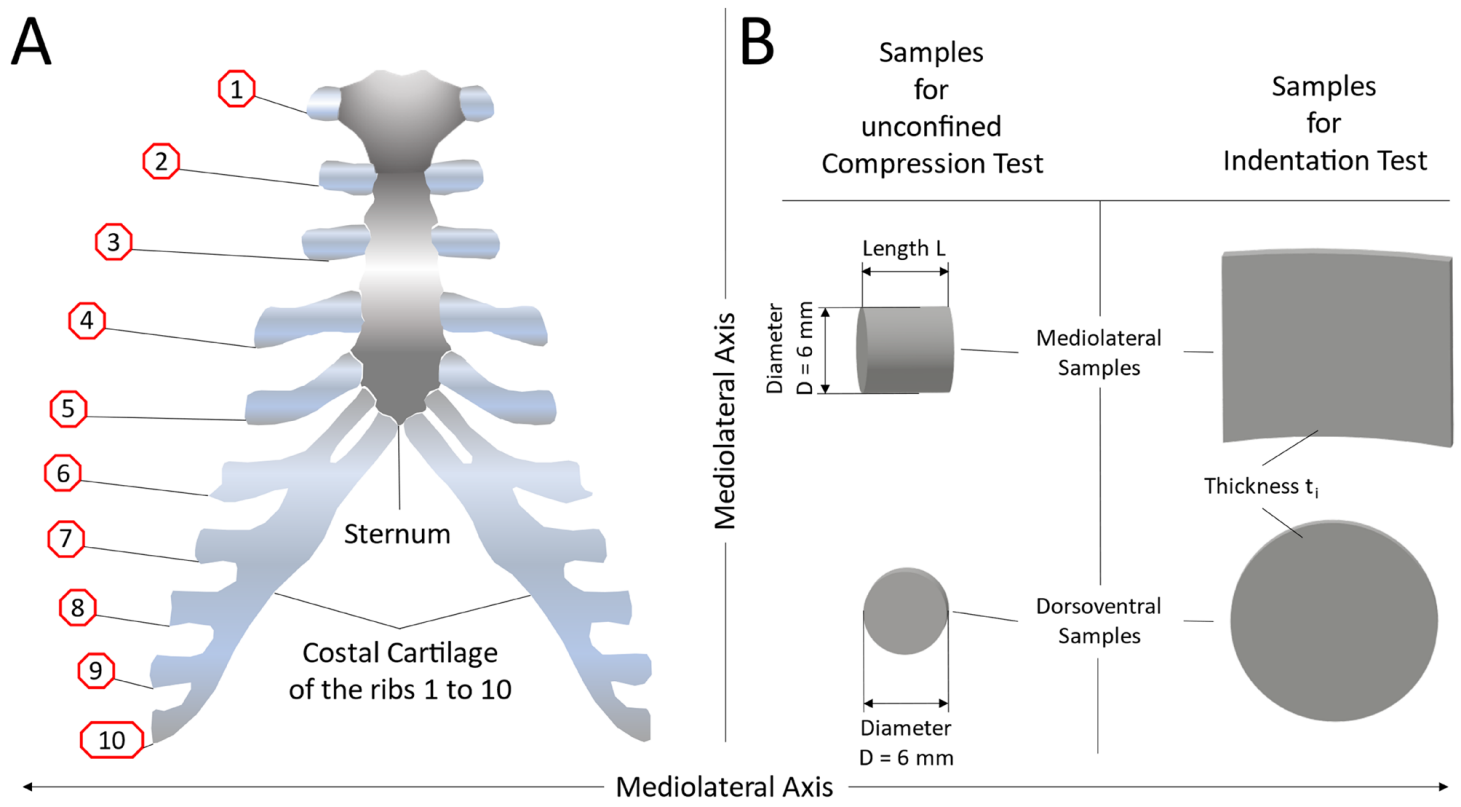
Sample harvesting of costal cartilage. (**A**) Schematic of the numbering of the individual rib location. (**B**) Schematic of the samples for the unconfined compression test and the indentation test in mediolateral and dorsoventral direction.

Due to the non-availability in obtaining samples of costal cartilage from surgical procedures, research is limited. In this study, samples were harvested from frozen body donors and unfrozen homicide victims which influenced the number of bodies and age range of the samples. Other studies encountered similar difficulties concerning the availability of samples^13^. Costal cartilage was harvested from 12 cadavers, both male and female with an average age of 63 ± 24.8 years (Table 1). The samples were obtained from the rib cage (Fig. 1A) from every individual cartilage carrying rib (Rib No. 1 to 10) as far as the dimensions of the cartilage and the grade of calcification allowed it. All samples were taken equally in the middle between the sternum and the rib bones and only of the midsubstance of the cartilage without the perichondrium. Afterwards, the cartilage was cut into preliminary samples, wrapped in saline-soaked (NaCl 0.9%) gauze, overwrapped with aluminum foil and stored at − 20 °C until tested^28^. Prior to testing, the preliminary samples were immersed in saline (NaCl 0.9%), thawed for at least 45 min to ensure full thawing and stress equilibration and cut into final shape. The cutting orientations were chosen to provide samples to be tested in dorsoventral and mediolateral orientation (Fig. 1B).

**Table 1:** Demographic variables of the cadavers and sample information.

Body	Sex	Age	Compression			Indentation				
			Ribs	Dorso-ventral	Medio-lateral	Ribs	Dorso-ventral		Medio-lateral	
				No. of Samples	No. of Samples		No. of Samples	No. of Tests	No. of Samples	No. of Tests
1	m	85	None	None	None	R6, R7, R8, R9	4	12	7	18
2	f	78	R7, R8, R9, R10	7	7	R5, R6, R7, R8, R9, R10	14	37	17	46
3	f	75	R1, R2, R3, R4, R5, R6, R7, R8, R9	11	20	R1, R2, R3, R4, R5, R6, R7, R8	14	37	20	57
4	m	79	R7, R8, R9	2	6	R4, R5, R7, R8, R9, R10	6	20	15	37
5	f	76	R2, R6, R7	1	6	R2, R3, R4, R6, R7, R8, R9	12	35	12	33
6	f	94	R2, R6, R7	1	6	R7, R8	2	6	6	18
7	m	27	R2, R3, R4, R5, R6, R7, R8	18	24	R2, R3, R4, R5, R6, R7, R8	16	42	17	45
8	f	29	R2, R3, R4, R5, R6, R7	10	17	R2, R3, R4, R6, R7, R8, R9	12	32	14	34
9	f	32	R2, R3, R4, R5, R6, R7	12	15	R2, R3, R4, R5, R6, R7, R8	16	46	18	52
10	f	79	None	None	None	R2, R3, R4, R6, R7, R9	9	27	14	39
11	m	68	R5, R6, R7, R8, R9	12	11	R6	1	3	3	9
12	f	34	R8, R9	6	6	R4, R5, R6, R7, R8	15	40	15	42
Total = 12	∑ f = 8 ∑ m = 4	63 ± 24.8 (27 – 94)	∑ = 48	∑ = 80	∑ = 118	∑ = 66	∑ = 121	∑ = 337	∑ = 158	∑ = 430

R = number of individual rib where sample was harvested; m = male, f = female; mean value (± standard deviation; minimum—maximum) of age is presented at the end of the table; ∑ = Sum.

### Unconfined compression test

**Figure 2:**
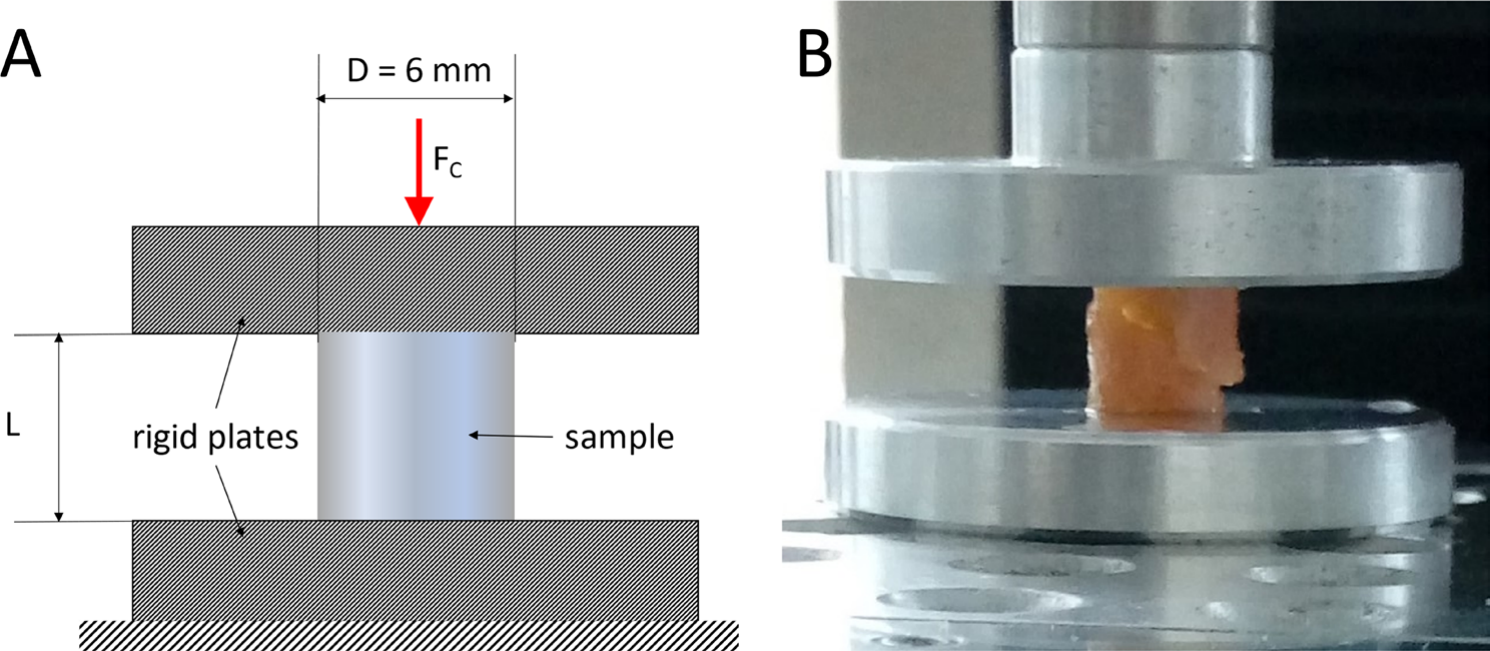
Experimental set-up of the unconfined compression test. The cylindrical samples were placed between two rigid plates (aluminum) with high stiffness. The friction between plates and specimen was reduced by lubricating the specimens and both plates with NaCl 0.9% before testing. (**A**) Schematic test set-up. (**B**) Photographic test set-up. The figure shows a sample after the test has been performed. The sample has a diagonal crack from the upper left corner to the lower right.

For this test, cylindrical samples were punched out of the specimen of cartilage using a bone graft harvester (Arthrex AR-1981-06H) with a diameter of 6 mm and afterwards cut to length L = 5.9 ± 0.7 mm (N = 198) manually using a scalpel. The length L to diameter d ratio was chosen to get slenderness ratios λ below 6 (λ_min_ = 2, λ_mean_ = 3.3, λ_max_ = 5.3, Eqs. 1 to 4) to prevent the samples from buckling^29^. The length L was measured using a caliper. The unconfined compression test (Fig. 2) was performed at room temperature, using a desktop-type, single-column universal materials testing machine (Zwick BZ2.5/TN1S) with a 2.5 kN force sensor. A preloading of 0.1 N was applied with a velocity of 0.05 mm/s. Next, the indentation depth and force were reset and the sample was compressed with a velocity of 1.0 mm/s until failure. Time, force and displacement were measured at a sampling rate of 50 Hz. The data was imported into MATLAB (R2018a) and the compression modulus of the sample was calculated in fitting a class 5 polynomial to the discrete stress–strain values. The point of constant slope of the polynomial was calculated by determining the maximum value of the first derivative. The Young's Modulus for compression $E_{c}$ is defined as the slope of the linear part of the stress–strain curve. The compressive strength was measured according to Eq. (5).

1 $$A = \frac{{\pi \cdot d^{2} }}{4}$$

Equation (1) denotes Cross-section A of the sample in the initial state; sample diameter d

2 $$I = \frac{{\pi \cdot d^{4} }}{{64}}$$

Equation (2) denotes Smallest axial inertia moment I

3 $$i = \sqrt {\frac{I}{A}}$$

Equation (3) denotes Radius of inertia i

4 $$\uplambda = \frac{{\beta \cdot L}}{i}\;{\text{with}}\;~\beta = 1\;{\text{for}}\;{\text{Euler}}\;{\text{buckling}}\;{\text{case}}\;{\text{II}}$$

Equation (4) denotes Slenderness ratio λ, sample length L, β coefficient of buckling length

5 $$\sigma = \frac{F}{A}$$

Equation (5) denotes Compressive strength $\sigma$, Load applied F, specimen area A.

### Indentation test

**Figure 3:**
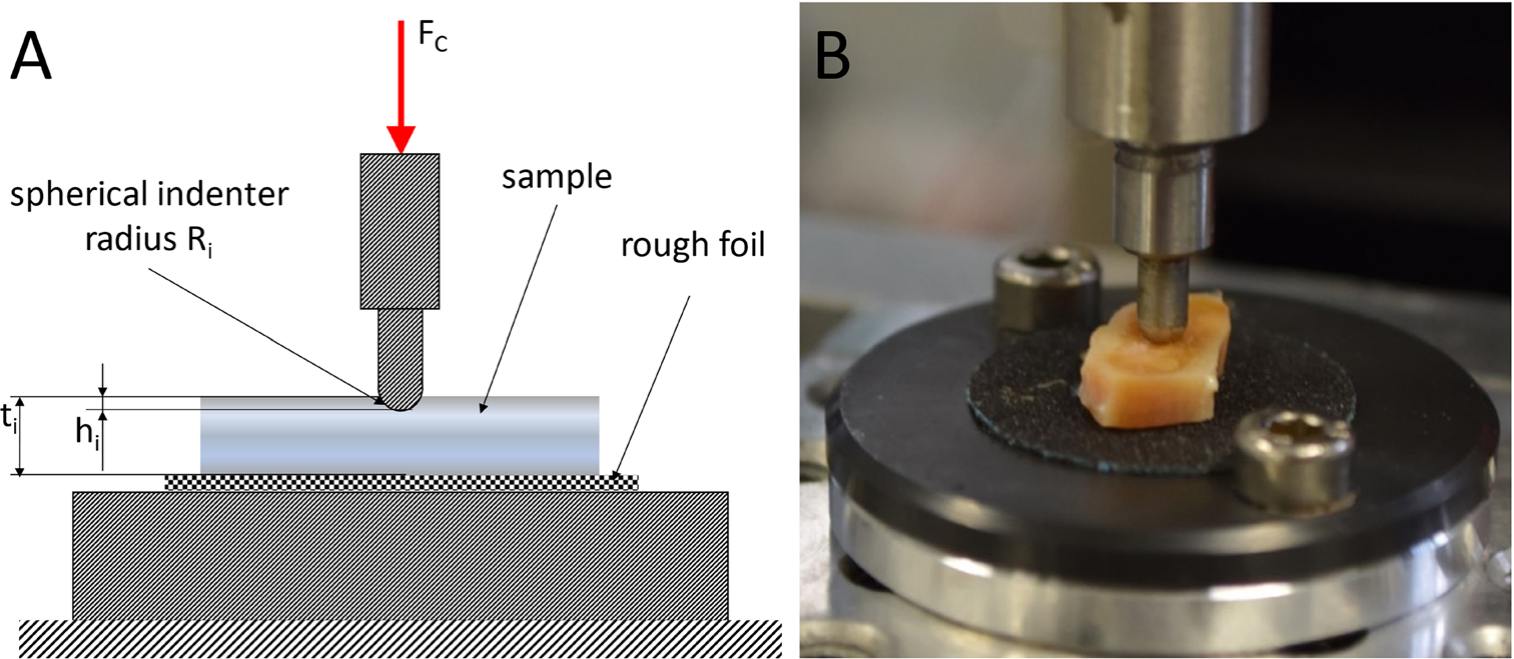
Experimental set-up of the indentation test. As indenter a custom made rigid spherical-tip (steel, radius R_i_ = 1.5 mm) was used. The sample slices were centered below the indenter on a rigid plate. The surface of the plate was covered with a rough foil to prevent the samples from slipping. (**A**) Schematic test set-up. (**B**) Photographic test set-up.

For the indentation test, specimen slices were manually cut from the cartilage with scalpels. The thickness t_i_ = 5.7 ± 1.7 mm (N = 279) of the specimens was measured using a caliper. The indentation testing was performed at room temperature, using a desktop-type, single-column universal materials testing machine (Zwick BZ2.5/TN1S) with a 100 N force sensor (Fig. 3). To reduce friction between the contact surface of the indenter and the sample, the cartilage was moistened with NaCl 0.9%. A preload of 0.1 N was applied with a velocity of 0.05 mm/s. Next, the indentation depth and force were reset and the indenter was lowered with a velocity of 0.5 mm/s to the maximum indentation depth h_max_ of 0.9 mm. Time, force and displacement were measured at a sampling rate of 50 Hz. The data was imported into MATLAB (R2018a) and the Young's Modulus of indentation $E_{i}$ was calculated (Eq. 6) where F is the Force, $\upnu$ is the material Poisson’s ratio ($\upnu ~$ = 0.5 for an incompressible solid^13,30,31^), R is the radius of the indenter (R = 1.5 mm) and h_i_ is the depth of indentation. Depending on the sample thickness, $E_{i}$ was calculated for each sample at a strain of 5% (E_i5%_) 10% (E_i10%_) and at the maximum strain (E_imax_) thus maximum indentation. The strain was calculated as the quotient of the sample thickness and the corresponding penetration depth. To avoid mutual interference of the indentations, a distance of at least three times the indenter radius was maintained between the indentations and between the sample edge and the indentations. For verification, the values of the 1st, 2nd, 3rd and 4th test impressions were compared with each other by using the Mann–Whitney-U test. No significant differences were found (*P* > 0.05).

6 $$E_{i} = \frac{3}{4}F\frac{{1 - \nu ^{2} }}{{\sqrt R \cdot h_{i}^{{\frac{3}{2}}} }}$$

Equation (6) denotes Young's Modulus of indentation for the isotropic elastic Hertzian contact of a rigid spherical indenter and an incompressible material^32–34^.

### Histology

Samples from the costal cartilage of four bodies (29, 34, 79 and 94 years) were taken along the frontal and sagittal planes for histology. The tissue was fixed in 10% buffered formalin for 24 h and afterwards decalcified in 0.5 M ethylenediaminetetraacetic acid (EDTA), depending on the degree of calcification, for about 5–8 days. The samples were then cut to a thickness of 5 mm and embedded in paraffin. Sections with a thickness of 3 µm were prepared with a sliding microtome and dried at 37 °C. The sections were then dewaxed and treated with picrosirius red for collagen bundle staining^35–38^. The microscopic examination was performed using crossed polarized filters (Keyence VHX-2000; Osaka, Japan).

### Statistics

All statistical analyses were performed using IBM SPSS Statistics Version 25 (IBM Corp.). A *P*-value below 0.05 was considered as statistically significant. All indentation and unconfined compression test data were checked with the Kolmogorov–Smirnov and Shapiro–Wilk tests and are not normally distributed (both *P* < 0.05). To make the results comparable to previous studies, all results are presented as mean values ± standard deviation. In addition, Table 2 shows the results per body donor as median and interquartile ranges. The Mann–Whitney-U-Test for independent samples was conducted to determine the influence of sex, age and origin of the samples. Pearson correlation was calculated to test the influence of age. Anisotropy and the influence of rib position was investigated using the Wilcoxon test for dependent samples.

**Table 2:** Overview of all results per body, given as median and interquartile ranges (IQR).

Body	Sex	Age	Compression				Indentation			
			E_C_		E_i5%_		E_i10%_		E_imax_	
			50%	IQR	50%	IQR	50%	IQR	50%	IQR
7	m	27	26.91	19.12	13.57	6.08	15.65	6.55	16.24	7.75
8	f	29	49.97	17.6	14.87	8.32	18.47	11.95	18.95	16.02
9	f	32	32.57	23.46	13.02	7.94	14.93	8.92	17.66	9.75
12	f	34	26.5	28.5	13.05	10.31	15.72	11.73	15.75	10.46
11	m	68	31.26	19.75	15.05	8.92	17.36	8.95	17.91	9.77
3	f	75	16.27	18.69	10.12	4.64	12.11	5.55	15.14	7.29
5	f	76	30.11	27.14	8.03	5.88	10.88	6.87	12.01	6.39
2	f	78	13.98	15	6.52	4.6	7.77	5.88	7.72	5.9
4	m	79	22.45	29.62	8.9	3.74	10.77	6.41	12	6.64
10	f	79	None	None	8.52	4.83	9.75	5.64	11.83	7.02
1	m	85	None	None	7.95	3.34	9.59	5.3	9.93	6.73
6	f	94	36.25	31.13	5.35	3.05	6.86	3.34	6.83	3.32

The table is ordered by the age of the body donors. E_i5%_, E_i10%_, E_imax_ are the Young's Moduli of indentation at a strain of 5%, 10% and at the maximum strain (Eimax). E_C_ is the Young's Modulus of compression.

### Ethics declaration

The samples used in this study were obtained according to the ethical guidelines approved by the Ethics Commission of Cologne University’s Faculty of Medicine (Application No. 18-220). The body donor had provided informed consent while alive for the use of her body for medical, scientific, and educational purposes at the Anatomical Institute of the Medical Faculty, University of Cologne, Germany. The samples from the homicide victims were collected for forensic examinations ordered by the public prosecutor's office. Informed consent from the next of kin for these sample donors was not required by the approving ethics committee because it was a legal acquisition.

## Results

### Youngs Moduli and compressive strength

The unconfined compression test resulted in a mean Young’s Modulus of E_c_ = 32.9 ± 17.9 MPa and a mean compressive strength of $\sigma$ = 6.1 ± 3.0 (all N = 10). The indentation testing yielded Young’s Moduli of E_i5%_ = 11.1 ± 5.6 MPa, E_i10%_ = 13.3 ± 6.3 MPa and E_imax_ = 15.6 ± 6.6 MPa (all N = 12).

### Sex and age effects

We could not detect a sex-specific effect in the results of the unconfined compression test or the indentation test (both *P* > 0.05).

**Figure 4:**
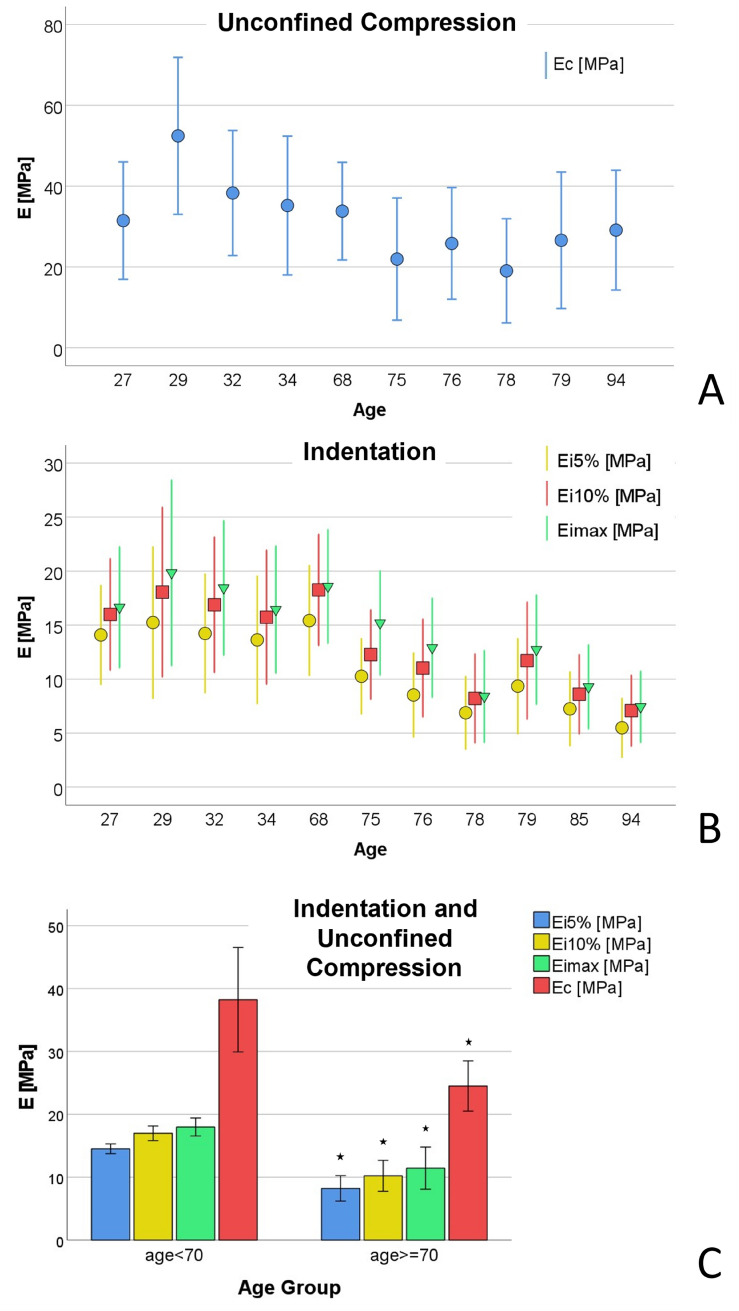
The effect of age. (**A**) Distribution of the Mean Young’s Modulus for the unconfined compression test E_C_ over the age. (**B**) Distribution of the Mean Young’s Modulus for the indentation test E_i5%_, E_i10%_, E_imax_ over the age. (**C**) Mean Young’s Moduli E_C_ ± SD, E_i5%_ ± SD, E_i10%_ ± SD, E_imax_ ± SD per age group (age < 70 years, age ≥ 70 years) with *significantly higher results for the group of the ≥ 70 years old cadavers (*P* = 0.009 for E_C_, *P* = 0.004 for E_i5%_, E_i10%_, E_imax_).

When looking at the distribution of the results of both tests a decrease in the values of the Young’s Modulus was found with increasing age (Fig. 4A,B). Age and Young’s Moduli were found to be moderately negatively correlated (E_C_: *r* = − 0.37, *p* < 0.001; E_i5%_: *r* = − 0.51, *p* < 0.001; E_i10%_: *r* = − 0.48, *p* < 0.001; E_imax_: *r* = − 0.46, *p* < 0.001).

Two groups were formed (age group 0 < 70 years, age group 1 >  = 70 years) with mean ages of 80 ± 6.1 years (N = 7) for group 0 and 38 ± 15.2 years (N = 5) for group 1 and a strong significant influence of age on all results was found for E_C_ (*P* = 0.009) and E_i5%_, E_i10%_, E_imax_ (all *P* = 0.004, Fig. 4C).

### Anisotropic behavior

**Figure 5:**
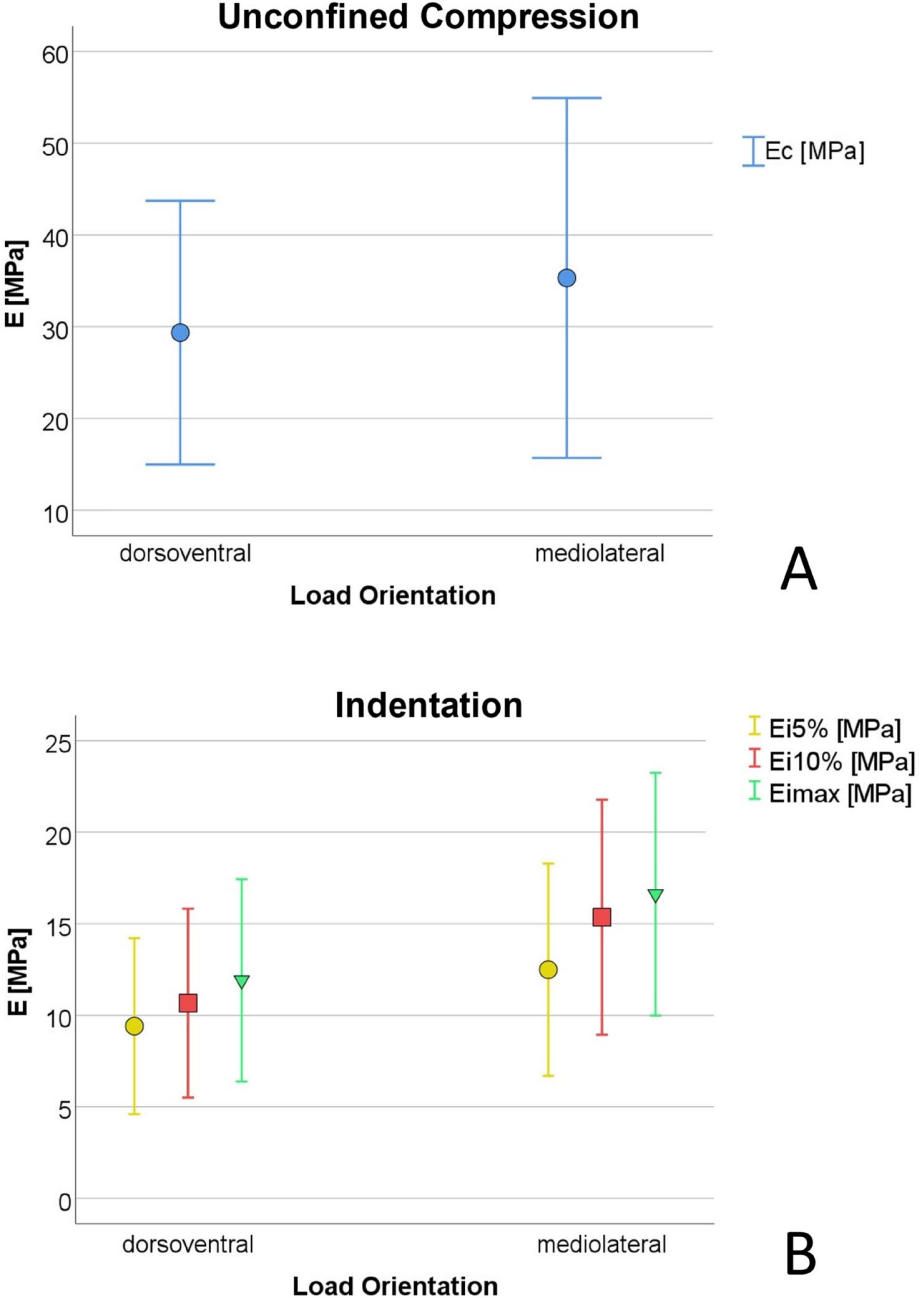
The effect of the load orientation on the Mean Young’s Moduli for (**A**) the unconfined compression E_C_ ± SD and (**B**) indentation E_i5%_ ± SD, E_i10%_ ± SD, E_imax_ ± SD with *significantly higher results for the Young’s Moduli calculated in the indentation test (all *P* = 0.002).

**Figure 6:**
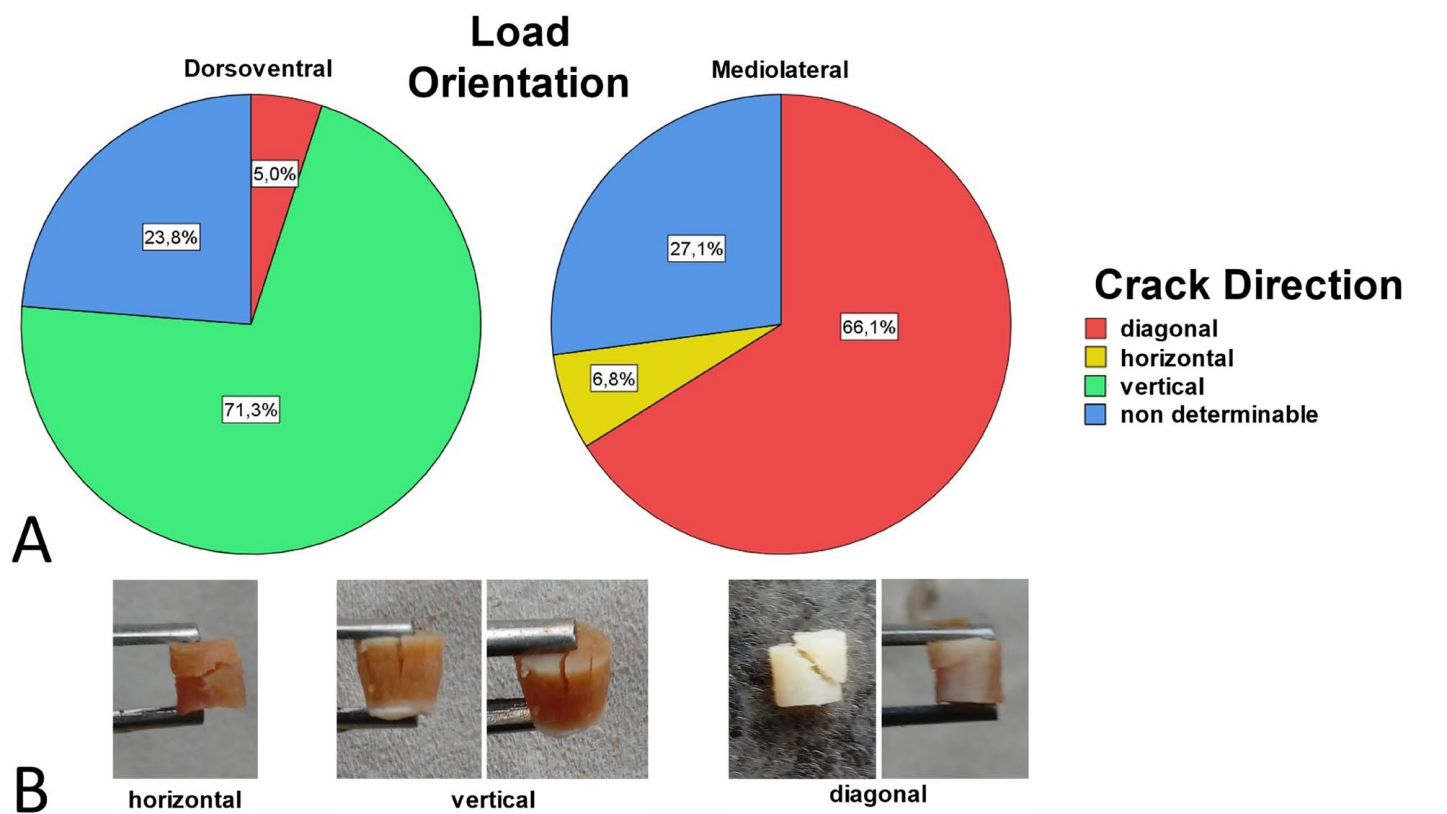
Direction of the crack at failure of the samples for the unconfined compression test. (**A**) The graph shows the percentage of samples broken diagonally, horizontally or vertically for each load direction, as well as those samples for which the crack direction could not be determined visually. (**B**) Examples of fractured samples.

The results of the unconfined compression test could not prove an effect of the test direction (dorsoventral and mediolateral) on the Young’s Modulus (*P* = 0.508, Fig. 5A) or the compressive strength (*P* > 0.05). For further analysis of the material behavior, the direction of the crack at failure of each sample of the unconfined compression test was determined (Fig. 6). While the majority of dorsoventrally loaded specimens fractured along the loading orientation, none of the mediolateral loaded specimens did. It is noteworthy that the dorsoventrally loaded and fractured specimens did not show a single crack in comparison to the mediolateral specimens, but several cracks in a fan-shaped structure, so that individual thick tissue bundles were visible.

A comparison of the results of the indentation test of the dorsoventrally and mediolaterally tested samples (Fig. 5B) showed a strong significant effect (*P* = 0.002) of the load orientation with in mediolateral direction higher Young’s Moduli compared to the dorsoventral direction (for E_i5%_ dorsoventral 8.8 ± 3.0 MPa, mediolateral 12.3 ± 4.3 MPa, for E_i10%_ dorsoventral 10.0 ± 3.3 MPa, mediolateral 15.1 ± 4.7 MPa, for E_imax_ dorsoventral 11.1 ± 3.5 MPa, mediolateral 16.2 ± 5.0 MPa).

### Individual costal cartilages

Where a sufficient number of samples were available, meaning that pairs could be formed, the results of each individual rib were compared statistically e.g. rib no. 1 versus 2. By analyzing the results of the unconfined compression test no significant differences could be detected (all *P* > 0.05). Pairing the results of the indentation test of every individual rib with each other revealed significant deviations for E_i5%_ for the combinations of rib 2 versus 3 (*P* = 0.046, N = 6), rib 2 versus 6 (*P* = 0.028, N = 6), rib 2 versus 7 (*P* = 0.046, N = 6) and rib 2 versus 8 (*P* = 0.043, N = 5), for E_i10%_ for the combinations of rib 2 versus 3 (*P* = 0.046, N = 6), rib 2 versus 6 (*P* = 0.028, N = 6) and rib 2 versus 7 (*P* = 0.046, N = 6). For all other pairings for E_i5%_ and E_i10%_ and for all pairings of E_imax_ no significant deviations could be detected.

### Histology

**Figure 7:**
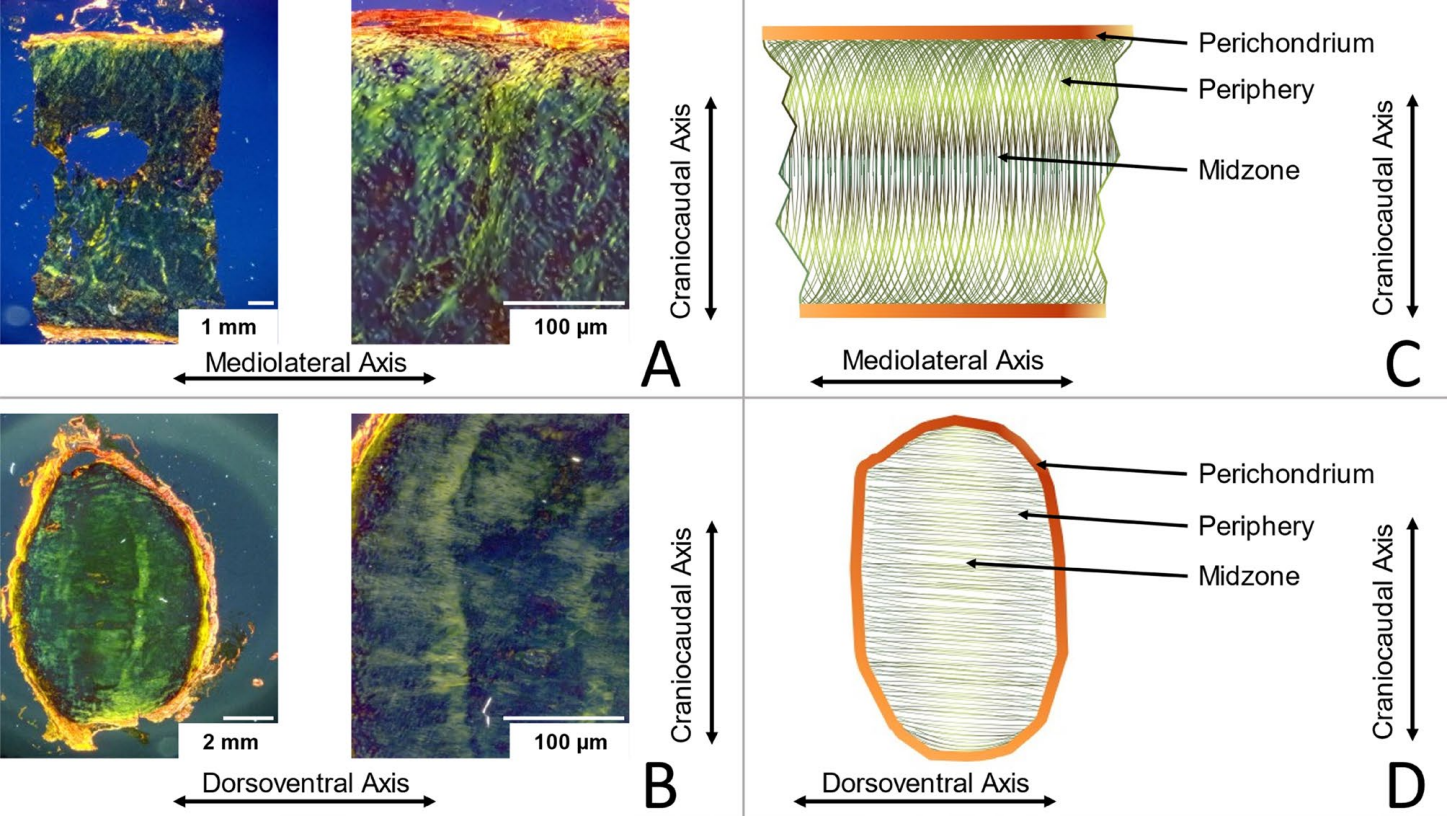
Costal cartilage samples stained with picrosirius red and examined with polarized light: (**A**) frontal plane, (**B**) sagittal plane. Schematic of the collagen fiber orientation (**C**) in frontal and (**D**) sagittal plane.

The histochemical examination showed preferred orientations of the collagen structure (Fig. 7). In the analysis of the frontal plane samples, the collagen structure in the midzone of the cartilage was mainly craniocaudally organized and showed in the periphery an arc-shaped pattern oriented towards the perichondrium. When examining the sagittal plane samples, the course of the collagen fibers in the midzone seemed to be more oriented along the dorsoventral axis.

## Discussion

To date, limited research has been performed on the elastic material behavior of human costal cartilage and there is a lack of knowledge about the direction-dependent material properties. In this study, we therefore analyzed the anisotropic material behavior of human costal cartilage and the influence of age and sex by evaluating the elastic moduli using indentation (E_i5%_, E_i10%_, E_imax_) and unconfined compression tests (E_C_).

We chose a rather slow preloading velocity of 0.05 mm/s to provide contact between indenter/plate and sample without the risk of exceeding the preload. For the actual tests, we applied the load in a more rapid manner to exclude the time-dependent part of the viscoelastic material behaviour leaving only the elastic material properties to be measured. This methodology differs from the comparable studies in the references, since the time-dependent material behaviour was also measured there. In regard to the computer-aided simulation of accident events and the resulting injuries 25–27 and for the forensic examination of tool marks on tissue the time dependent material properties need to be excluded and only the spontaneous elastic behaviour is relevant.

The Young’s Modulus for compression calculated in this study E_c_ = 32.9 ± 17.9 MPa (N = 10 cadaver, 198 samples) is about three times higher and shows a higher standard deviation than the results of Hendel and Lenk^10^ who calculated a Young’s Modulus of E_c_ = 9.1 ± 1.4 MPa (N = 90 cadaver, 90 samples) and about three times lower than the results of Grellman et al.^9^ who calculated a Young’s Modulus of E_c_ = 103.4 ± 30.1 MPa (N not given). One possible influence could be the use of different fluids to store the samples. While Hendel and Lenk^10^ fixed the samples in Románhyi-solution^39^ and Grellman et al.^9^ used Ringer's solution, we used saline (NaCl 0.9%) as it was done by Forman et al^18^. Oyen et al.^31^ performed indentation tests in porcine costal cartilage samples submerged in physiological saline and in water and found that the samples soaked in water showed an increase of tissue stiffness. Further studies are needed to investigate those large dimensioned deviations.

A reason for the relatively high standard deviation of our results in the unconfined compression test for E_c_ of ± 17.9 MPa could be found in the wide range of age of our samples (27–94 years). Many studies have shown, that calcification of the costal cartilage is age dependent^40–42^. Forman et al.^18^ performed bending tests on costal cartilage and suggested that calcification could have a substantial effect on the stiffness of the rib cage. Forman's results indicate an increase in local stiffness, whereas our results show a reduction in stiffness with increasing age. Further studies will be necessary to clarify this contradiction. The mean compressive strength calculated in this study of $\sigma$ = 6.1 ± 3.0 (all N = 10) is in good agreement with the results of Feng et al.^7^ who calculated a compressive strength of 6.3 ± 1.0 (all N = 28). Nevertheless, the deviation of the mean age of the subjects in this study (63 ± 24.8 years) from those in the work of Feng et al. with 4.4 years could be of relevance. Since Feng et al. examined tissues with an age range of 3 to 6 years and in this work 27 to 94-year-old bodies were examined, further studies are needed to fill those gaps.

Our results of the Young’s Moduli for the indentation tests (E_i5%_, E_i10%_, E_imax_) are considerably higher than the results of Lau et al.^13^ who performed indentation tests and yielded a mean Young’s Modulus of E_0_ = 5.2 MPa with a similar indenter (R = 1.575 mm) and strain (7%) but with a noticeably higher loading time of 2.125 s compared to 0.57 ± 0.17 s (E_i5%_,), 1.12 ± 0.31 s (E_i10%_) and 1.75 ± 0.12 s (E_imax_) in this study. We hypothesize that the longer time to measure the Young's modulus might influence the results. The biphasic properties of cartilage tissue cause fluid to leak during indentation, thereby reducing the stress in the tissue. The longer the loading phase until the indentation depth required for measurement is reached, the lower the measured Young’s Modulus. Nevertheless, it seems doubtful whether the difference in the values measured by Lau in this study can be explained by this alone. Further studies are needed to clarify the deviation. A possible approach would be to determine the reduction of stress in the material by measuring the amount of water that leaks out during the indentation.

When comparing the Young's Moduli calculated in the indentation test, it was found that E_i5%_ < E_i10%_ < E_imax_ (all *P* < 0.05). We hypothesize that due to the aforementioned rather short loading time the time-dependent components of the otherwise viscoelastic material behavior play a minor role. During the test the material behavior of the cartilage can be considered as approximately elastic. Under this assumption, the tissue shows the elastic properties of a spring with a progressive rate, which means that the modulus of elasticity increases with increasing elongation. Further studies are needed to calculate the exact purely elastic response of the costal cartilage.

Neither the indentation nor the unconfined compression tests showed a sex-specific effect (both *P* > 0.05, Fig. 4A) which supports the findings of Guo et al.^4^ who did not find a significant difference in tensile strength between males and females comparing all age groups. In consideration of the relatively limited number of bodies in this work, this result should be confirmed with further studies.

Our results did show a decrease of the Young’s Modulus with increasing age. The strength of these results is limited by the small number of samples. Further studies are necessary to confirm those findings. The results supports of Guo et al.^4^ showed a decrease in tensile strength with increasing age. However, the age distribution of their study is 5–25 years and thus lies beside the distribution of the work presented here (26–94 years). Nevertheless, our results support Guo et al.^4^ in so far as they also reveal an age dependence of the changes in material properties.

The results of the indentation test clearly demonstrate the anisotropic material behavior of cartilage tissue. Although the values for the Young's Modulus calculated in the compression test do not support this result, the clearly direction-dependent crack behavior of the samples indicates anisotropy of the cartilage. The histological examination of the organization of the collagen structure also provides good support for these findings. The dorsoventrally loaded samples of the unconfined compression test are fractured primarily along the main direction of the collagen structure, which in this case, corresponds to the loading direction. The collagen structure of the mediolateral loaded samples runs approximately perpendicular to the load orientation. These samples mainly fractured diagonal, and to a small percentage, in the horizontal direction. None of these samples fractured in the vertical direction, i.e. perpendicular to the collagen fiber orientation. It can therefore be assumed that the direction of the crack depends on the collagen structure and that a crack occurs oriented along the fiber structure and not perpendicular to it. The load on the cartilage tissue in the mediolateral direction is perpendicular to the course of the fibers found in the histological examination and presses the fiber bundles together. We hypothesize that this requires a higher force than when the tissue is loaded in the dorsoventral direction, since the tensile-stable fibers are compressed along their orientation and can therefore absorb less load. Jurvelin et al.^43^ suggest, that the permeability of the pores in the cartilage matrix has a form and/or orientation responsible for an anisotropic friction for water flow which leads to anisotropic material properties. It seems likely that the friction of the water depends on the loading velocity, so that the influence should be small in the quasi-static tests carried out here. However, it should be noted that when measuring a sample cut out of the body, the boundary conditions are reduced by the otherwise supporting effect of the surrounding tissue. Therefore, the material characteristics in situ may differ from those measured in this study.

There are a few limitations to be mentioned. The low number of cadavers available for this study consisted of body donors with a mean age of 81 years ± 7.2 (N = 6) and homicide victims with a mean age of 45 years ± 22.6 (N = 6). No cadavers of children and adolescents between the ages of 34 and 68 years were available for this study.

In our study we used samples harvested from frozen body donors (N = 6, 81.2 + /− 7.2 years) and unfrozen homicide victims (N = 6, 44.8 + /− 22.6 years), all stored at—20° C after harvesting. By comparing both groups a strong significant influence of the origin of the bodies was found for E_C_ (*P* = 0.000) and E_i5%_, E_i10%_, E_imax_ (all *P* = 0.000). Since both groups have also a significantly different average age (*P* = 0.000), it is not possible to distinguish whether the significantly different results are due to the different origin and treatment or the different ages of the bodies.

All tests in this study were performed at room temperature. Care should be taken when using the absolute values of the elastic moduli measured in this study for simulations, as the mechanical properties may differ at different temperatures, e.g. body temperature. A further limitation was the effects of calcification and ossification of the cartilage tissue. In the samples of several cadavers of older individuals, we found bony tissue in-growths and widespread calcification. While the indentation tests were able to bypass the areas that were strongly altered by ossification and calcification, this was not possible with the unconfined compression tests which could explain the comparatively large standard deviation in the results.

Forman et al.^44^ analyzed the role of the perichondrium to the mechanical properties of costal cartilage as a composite structure. In this work we have studied the material properties of the cartilage tissue itself without including the influence of the perichondrium.

By comparing the results for the mean Young´s Modulus of the indentation test for each individual rib we could only detect a significant deviation by the pairings of rib 2 with the ribs 3, 6, 7 and 8 for E_i5%_ and 3, 6 and 7 for E_i10%_ with lower results for rib 2. By analyzing all other possible pairings, we could not detect any significant effect. Further studies are needed to investigate the possibility of deviating material properties of the individual rib cartilages.

Based on the results of the compression test, no anisotropic material behavior could be confirmed. Nevertheless, the results of the indentation tests showed significant differences in mediolateral and dorsoventral directions. Those results were strongly supported by the direction of crack of the compression test samples and the organization of the collagen structure of the cartilage revealed in the histological examination of the tissue. In summary, our results support the hypothesis that human costal cartilage has anisotropic material behavior.

Furthermore, we could show that there is a considerable age effect on the elastic material properties of the costal cartilage and that no significant sex effect exists.
